# One Size Doesn't Fit All - RefEditor: Building Personalized Diploid Reference Genome to Improve Read Mapping and Genotype Calling in Next Generation Sequencing Studies

**DOI:** 10.1371/journal.pcbi.1004448

**Published:** 2015-08-12

**Authors:** Shuai Yuan, H. Richard Johnston, Guosheng Zhang, Yun Li, Yi-Juan Hu, Zhaohui S. Qin

**Affiliations:** 1 Mathematics & Computer Science Department, Emory University, Atlanta, Georgia, United States of America; 2 Department of Biostatistics and Bioinformatics, Rollins School of Public Health, Emory University, Atlanta, Georgia, United States of America; 3 Department of Genetics, Department of Biostatistics, Department of Computer Science, University of North Carolina, Chapel Hill, Chapel Hill, North Carolina, United States of America; University of Canterbury, NEW ZEALAND

## Abstract

With rapid decline of the sequencing cost, researchers today rush to embrace whole genome sequencing (WGS), or whole exome sequencing (WES) approach as the next powerful tool for relating genetic variants to human diseases and phenotypes. A fundamental step in analyzing WGS and WES data is mapping short sequencing reads back to the reference genome. This is an important issue because incorrectly mapped reads affect the downstream variant discovery, genotype calling and association analysis. Although many read mapping algorithms have been developed, the majority of them uses the universal reference genome and do not take sequence variants into consideration. Given that genetic variants are ubiquitous, it is highly desirable if they can be factored into the read mapping procedure. In this work, we developed a novel strategy that utilizes genotypes obtained *a priori* to customize the universal haploid reference genome into a personalized diploid reference genome. The new strategy is implemented in a program named RefEditor. When applying RefEditor to real data, we achieved encouraging improvements in read mapping, variant discovery and genotype calling. Compared to standard approaches, RefEditor can significantly increase genotype calling consistency (from 43% to 61% at 4X coverage; from 82% to 92% at 20X coverage) and reduce Mendelian inconsistency across various sequencing depths. Because many WGS and WES studies are conducted on cohorts that have been genotyped using array-based genotyping platforms previously or concurrently, we believe the proposed strategy will be of high value in practice, which can also be applied to the scenario where multiple NGS experiments are conducted on the same cohort. The RefEditor sources are available at https://github.com/superyuan/refeditor.

This is a PLOS Computational Biology Software Article.

## Introduction

Mapping short reads onto the reference genome is a fundamental step in analyzing next generation sequencing (NGS) data and has been an area of intensive research in the past years. A wealth of successful software programs for mapping short reads, such as MAQ [1], SOAP [2], SOAP2 [3],BOWTIE [4], BOWTIE2 [5], BWA [6], BFAST [7], mrFAST [8], mrsFAST [9], NovoAlign (http://novocraft.com), SHRiMP [10], and STAR[11], have been developed and enjoyed wide-spread usage in many different NGS applications (e.g., whole genome sequencing (WGS) [12], whole exome sequencing (WES) [13], Chromatin Immunoprecipitation sequencing (ChIP-seq) [14–16] and transcriptome sequencing or RNA-seq [17]). The details of these programs can be found in excellent review articles [18, 19]. Despite the vast differences in algorithms and indexing methods, almost all of the existing read-mapping programs rely on the universal haploid reference genome—the National Center for Biotechnology Information (NCBI) human reference genome [20], which was derived from a small number of anonymous donors. At any multi-allelic position, a presumed consensus allele is used. Although carefully annotated and maintained, this single reference genome is not intended to represent all the variants found in the general population. Indeed, the human genome is diploid, and each individual possesses a unique set of genetic variants at millions of loci that distinguish him or her from others. Such wide-spread genetic variants, compounded with non-ignorable sequencing errors and short read length, cause a large proportion of reads to be unmapped or mapped to incorrect genomic locations. These mapping artifacts sometimes lead to misinterpretation of the NGS experimental results, such as the overstating the incidence of Allele Specific Expression [21–25] and affecting regulatory element identification at heterozygous variants [22, 26, 27].

Notably, genotype information is often available for samples that are undergoing NGS experiments. There are at least three scenarios in which the genotypes are available. First, many WGS or WES studies were conducted on samples that have been studied in the previous wave of genome-wide association studies (GWAS). These samples have already been genotyped by one of the array-based high-density genotyping platforms such as those from Illumina (San Diego, CA) and Affymetrix (Santa Clara, CA) [28]. Comprehensive assessment of array-based genotyping platforms can be found in the review article [29]. Second, many NGS experiments were conducted on well-established cell lines such as HeLa and IMR90, whose genotypes have also been profiled using array-based genotyping or resequencing. Third, and more often, array-based genotyping and multiple NGS-based experiments such as RNA-seq, ChIP-seq and resequencing were conducted on the same samples in the same study [30].

Using array-based genotyping, we will be able to collect genotype information on a large proportion of common genetic variants. Aided by powerful genotype imputation techniques, such as MaCH [31, 32], MaCH-Admix [33], IMPUTE [34], IMPUTE2 [35], Minimac [36] and BEAGLE [37], we will gain substantial additional genotype information on genetic variants that are not found on the genotyping array but are included on one of the dense reference haplotype panels such as those from the 1000 Genomes Project [12]. All of the aforementioned imputation methods exploit the linkage disequilibrium between observed and unobserved SNPs to infer the genotype of unobserved SNPs.

We believe that the substantial pre-existing genotype information, whether assayed or imputed, can be and should be utilized to fine tune the reference genome to reflect the unique features of each individual genome. An accurate reference genome sequence will lead to improved read mapping and consequently improved variant discovery and genotype calling.

**Fig 1:**
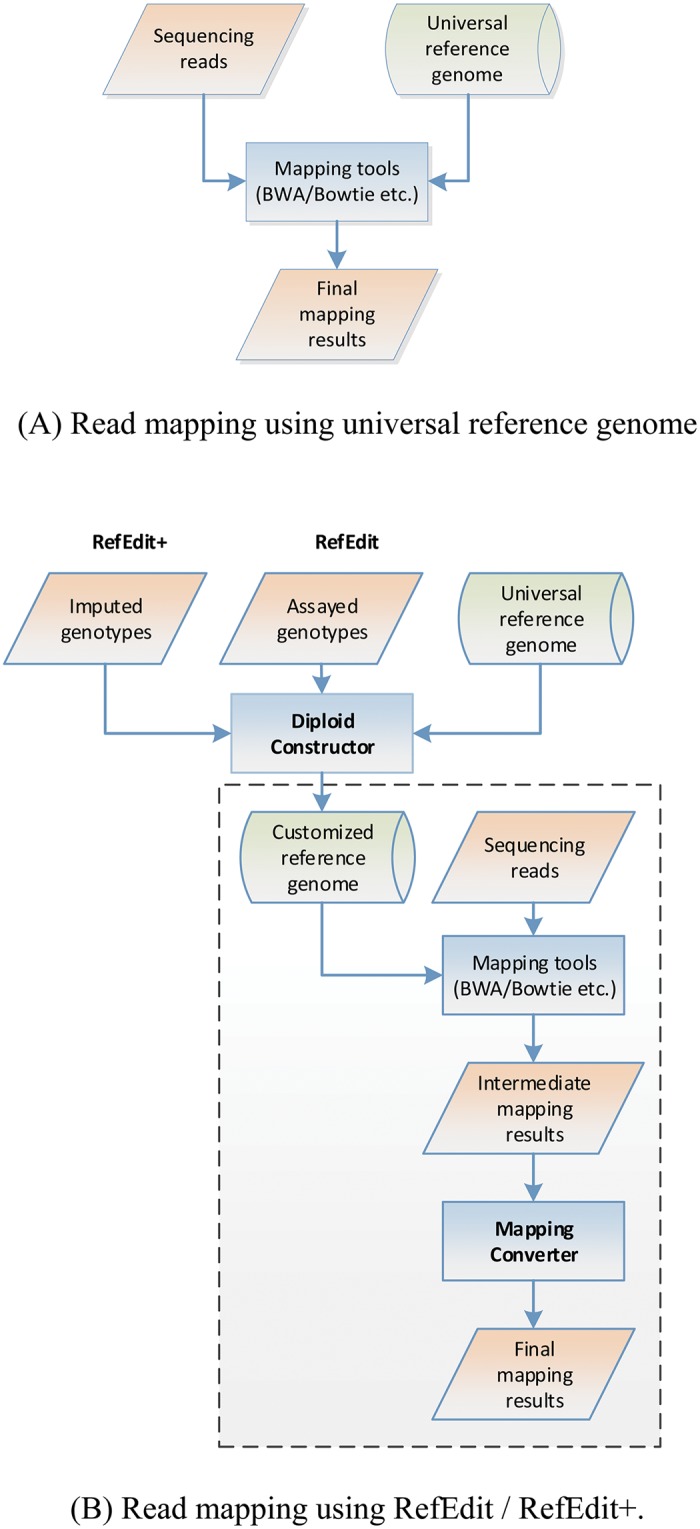
The pipeline for imputation, diploid reference genome construction and read mapping. (A) Traditional read mapping method. (B) RefEditor read mapping strategy that incorporates assayed and imputed genotypes.

Here we present RefEditor, a software package developed to improve read mapping by customizing the universal haploid reference genome to reflect individual genetic variation. It contains two components, RefEdit and RefEdit+, both converting the universal reference genome into a personalized diploid reference genome. RefEdit uses the assayed genotypes only whereas RefEdit+ adopts an additional step to augment the assayed genotypes by imputation. Fig 1 shows the comparison between standard read mapping process (Fig 1A) and the proposed read mapping process of RefEdit and RefEdit+ (Fig 1B). Both RefEdit and RefEdit+ contain two main components: Diploid Constructor and Mapping Converter. Diploid Constructor converts the universal haploid reference genome to the personalized diploid reference genome by supplementing the universal reference chromosomes with short sequences containing alternative alleles. Mapping Converter modifies intermediate results of read alignment in SAM (Sequence Alignment/Map) format [38] by translating mapped locations on customized, diploid reference genome back to its genomic locations on the universal reference genome and reassigning mapping quality scores. Diploid Constructor and Mapping Converter are called upon before and after executing the read alignment tools, respectively. More details about these steps can be found in the Methods section.

The idea of modifying the universal reference genome to accommodate genotype differences has been proposed in the literature before [22–25, 27]. These ideas, however, were developed under different context, mostly for reducing allele-specific mapping bias and are mainly used in RNA-seq and ChIP-seq studies. To the best of our knowledge, we are the first to apply personalized reference genomes to WGS data to assist genotype calling. We believe this is particularly important for two reasons. First, accurately identifying sequence variants is the basis of many population-based genetic studies. Second, many of the samples used in WGS or WES have previously been genotyped by array. These assayed genotypes should be utilized and our method enables that.

## Results

### Illustration of read mapping with personalized diploid reference genome

**Fig 2:**
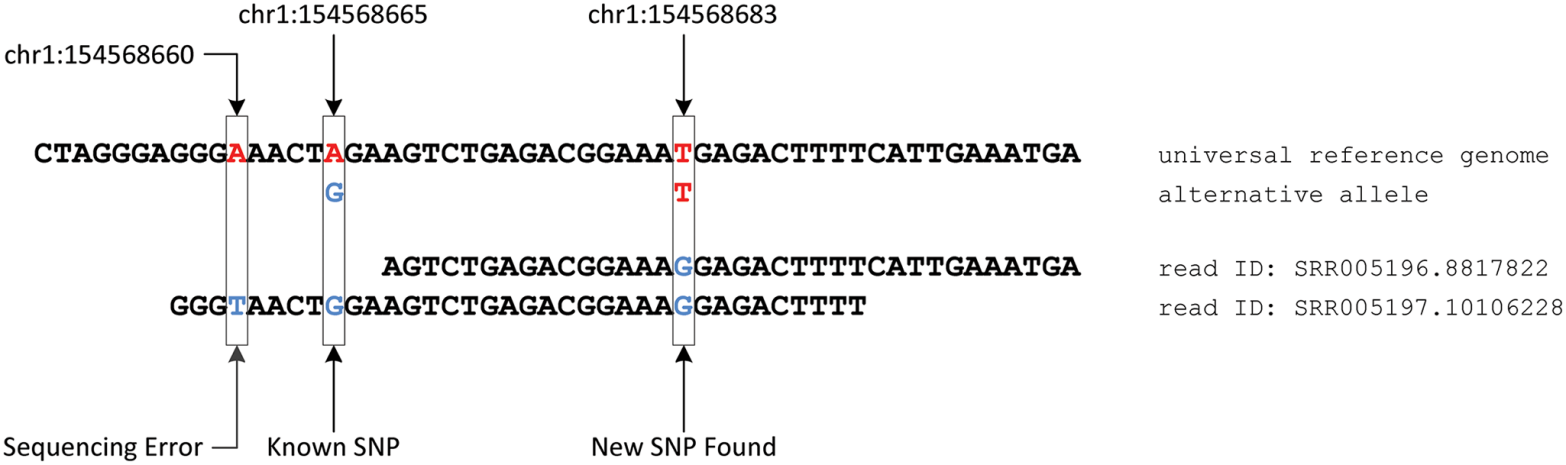
An example of how our RefEdit method can identify new variants from assayed genotypes. The maximum mismatch threshold is set to 2 by default. The assayed genotype is A/G at chr1:154568665. The read with ID SRR005196.8817822 is mapped to chr1:154568668 with 1 mismatch at chr1:154568683. The read with ID SRR005197.10106228 is mapped to chr1:154568657 of the alternative allele in the customized reference genome with 2 mismatches (chr1:154568660 and chr1:154568683). RefEdit discovers the new SNP at chr1:154568683 because of multiple existences of non-reference alleles. The Universal method, however, fails to map the read with ID SRR005197.10106228 because it exceeds the maximum mismatch threshold, therefore the new SNP cannot be discovered with confidence.

In Fig 2, we illustrate how including assayed genotypes improves the read mapping quality and SNP calling accuracy in a specific case using the sequence data from the 1000 Genomes Project. At the locus chr1:154568665, the reference allele is *A*. The sequencing read (ID: SRR005197.10106228) containing the alternative allele *G* at that locus can be successfully mapped to the personalized diploid reference genome with two mismatches. By contrast, this read fails to map to the universal reference genome because there are three mismatches, which exceeds the limit adopted by most mapping tools for this read length (36 base pairs (bp)). Downstream 18 bp at the locus chr1:154568683, multiple mapped reads show the same type of mismatch, suggesting that there might be a new SNP at that locus. The alternative allele *G* is not known *a priori*. This new SNP is verified by gold standard genotype calls based on Complete Genomics Inc. (CGI) deep sequencing data.

### Performance comparison study design

We conducted a series of studies using real data to evaluate the performance of RefEdit and RefEdit+ for read mapping, variant finding and genotype calling. In the first study, we focused on mapping success rates, genotype calling consistency and variant detection rates for two individuals from different populations. In the second study, we used Mendelian inconsistency (MI) among individuals in a trio as the metric for performance. In the third study, we used a different genotype gold standard to evaluate genotype calling consistency.

### Study samples

We selected samples from the HapMap [39, 40] and 1000 Genomes Project [12, 41] requiring that the samples have undergone both array-based genotyping and deep sequencing. Specifically, the African descent NA19238 and the European descent NA12716 were qualified and chosen for the first study, the African trio (NA19238 (mother), NA19239 (father) and NA19240 (child)) was chosen for the second study, and the European descent trio (NA12892 (mother), NA12891 (father) and NA12878 (child)) for whom phased haplotype information is available, was chosen for the third study.

### Genotypes from genotyping arrays

We chose the Affymetrix Axiom series array as the array-based genotyping platform in this study. This array contains about 6 million SNPs. We used the genotypes produced by the 1000 Genomes Project, which were called based on the CGI deep sequencing data, as the gold standard. This sequencing platform discovered about 41 million SNPs among 433 individuals. Both platforms produce high quality genotype calls and have been frequently used in other studies [42–44].

### Genotype summary from genotyping array and imputation

We use population-specific reference panels from the 1000 Genomes Project [41] for imputation. The panel consists of 246 African haplotypes and 379 European haplotypes. The reference panels we used do not contain haplotypes from the two trios we studied, neither do they contain haplotypes from offspring in any trio. To avoid biased results, the two haplotypes from the study sample (if present) are also excluded from the panel during each run.

It is of interest to know, from the existing array-based genotype data, how many genotypes containing the alternative allele are identified and how many more can be identified by genotype imputation. Genotype summaries (ref/ref, ref/alt, alt/alt proportions) for NA 19238 and NA 12716 are displayed in S1 Fig (also see S1 Table for numerical result). We used genotypes inferred from CGI sequencing conducted and reported by the 1000 Genomes project as the gold standard to evaluate the genotype concordance. A Venn’s diagram showing the overlaps between sets of assayed, imputed and the CGI gold standard genotypes can be found in S2 Fig. The Axiom genotyping platform has very high concordance in the overlapping part with CGI genotypes (99.75% for NA19238 and 99.83% for NA12716) as shown in the S2 Table, and hence is reliable. Details of the categorized consistencies between Affymetrix and CGI genotypes for individual NA19238 and NA12716 can be found in the S3 Table. The distribution of minor allele frequency (MAF) for genotyped and imputed SNPs are shown in S3 Fig.

The Rsq value is a good estimator of the correlation between the imputed and true genotypes, and thus is frequently used as a measure of imputation accuracy [32, 37, 45, 46]. By applying an appropriate Rsq threshold, we can achieve a reasonable balance between the number and the quality of imputed genotypes. The ratio of imputed genotypes that passed the threshold and their accuracy compared to CGI genotype at different Rsq thresholds can be found in the S4 Fig. We set the threshold at 0.7 to balance the number of qualified genotypes and quality of imputation, which retains 47.6% of the total imputed genotypes. The imputation accuracies for NA19238 and NA12716 are 99.05% and 99.32% respectively, as shown in the S2 Table. Details of the categorized consistencies between the imputed genotypes and the CGI genotypes for individual NA19238 and NA12716 can be found in the S4 Table. The numbers and proportions of newly imputed genotypes, along with those from the genotyping arrays, are shown in S1 Fig.

### Read mapping rate

Since we do not know the true genomic location of a sequencing read generated from real sequencing experiments, we are unable to directly compare mapping accuracy. The proportion of successfully mapped reads among all sequenced reads is a reasonable alternative, which had been used in other studies [47, 48]. A successful mapping is defined as a unique mapping with no more than two mismatches. Here we compared the numbers and proportions of successfully mapped reads using different read mapping approaches. In addition to RefEdit and RefEdit+, we included three additional mapping strategies: standard read mapping with universal reference genome, read mapping with ethnicity-specific major allele reference genome [48], and mapping with GSNAP (Genomic Short-read Nucleotide Alignment Program) [47].

**Fig 3:**
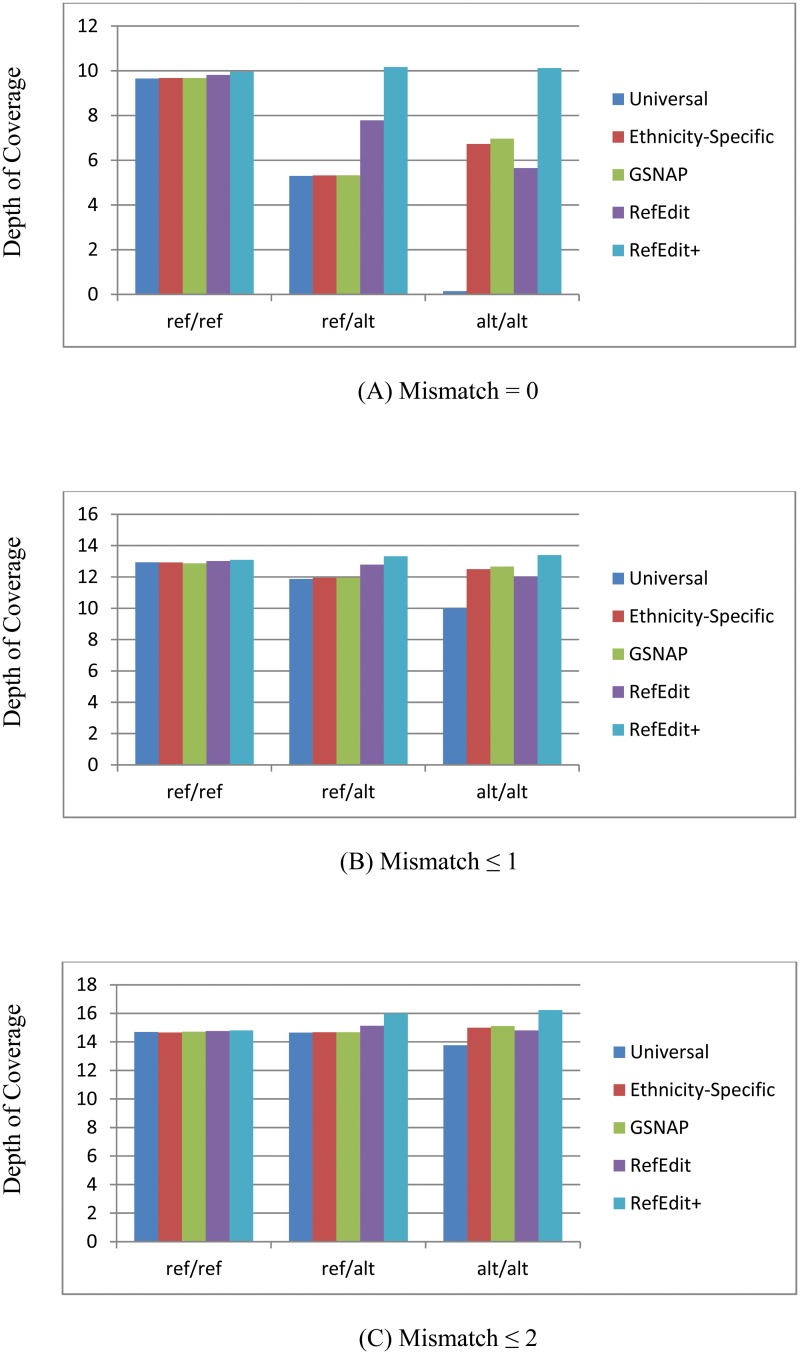
Average depth of mapped reads from the five mapping strategies for individual NA19238 (chr1~chr22), using CGI as gold standard for ref/ref, ref/alt and alt/alt loci. (A) Mismatch = 0. (B) Mismatch ≤ 1. (C) Mismatch ≤ 2. In the ref/ref loci group all methods have small differences in depth of coverage; in ref/alt and alt/alt groups RefEdit+ method shows much higher depth of coverage compared to other methods. The depths of coverage increase when maximum allowed mismatches increase.

Our results indicate that RefEdit and RefEdit+ methods show consistent improvement in terms of the read-mapping rate. Table 1 summarizes the mapping rates of five methods under three mismatch thresholds on individuals NA19238 and NA12716. Fig 3 shows the sequencing depth of mapped reads from the five mapping strategies at different genotype categories for individual NA19238 (chr1~chr22) using genotypes called from CGI sequencing data. Note that using the universal reference genome resulted in extremely low depth of coverage at alt/alt loci when no mismatch is allowed, which is expected because only reads with sequencing errors happening to match the reference allele can be mapped to those loci.

**Table 1:** Summary of read mapping rates of the five mapping strategies on individuals NA19238 (1,892,304,208 reads) and NA12716 (258,507,654 reads). The read length is 36 bp.

	**NA19238**		
	**Mapped reads**	**Difference**	**Mapping rates**
**Mismatch = 0**			
**Universal**	**762,614,756**	**0**	**40.30%**
**GSNAP**	**770,567,009**	**+7,952,253**	**+0.42%**
**Ethnicity-Specific**	**769,671,447**	**+7,056,691**	**+0.37%**
**RefEdit**	**776,314,807**	**+13,700,051**	**+0.72%**
**RefEdit+**	**789,080,981**	**+26,466,225**	**+1.40%**
**Mismatch ≤ 1**			
**Universal**	**1,020,457,855**	**0**	**53.93%**
**GSNAP**	**1,024,005,634**	**+3,547,779**	**+0.18%**
**Ethnicity-Specific**	**1,022,156,739**	**+1,698,884**	**+0.09%**
**RefEdit**	**1,026,574,577**	**+6,116,722**	**+0.32%**
**RefEdit+**	**1,032,966,073**	**+12,508,218**	**+0.66%**
**Mismatch ≤ 2**			
**Universal**	**1,158,462,316**	**0**	**61.22%**
**GSNAP**	**1,159,715,076**	**+1,252,760**	**+0.07%**
**Ethnicity-Specific**	**1,159,415,447**	**+953,131**	**+0.05%**
**RefEdit**	**1,162,647,233**	**+4,184,917**	**+0.22%**
**RefEdit+**	**1,167,809,214**	**+9,346,898**	**+0.49%**
		**NA12716**	
	**Mapped reads**	**Difference**	**Mapping rates**
**Mismatch = 0**			
**Universal**	**118,489,495**	**0**	**45.84%**
**GSNAP**	**120,156,152**	**+1,666,657**	**+0.64%**
**Ethnicity-Specific**	**119,600,653**	**+1,111,158**	**+0.43%**
**RefEdit**	**120,234,307**	**+1,744,812**	**+0.67%**
**RefEdit+**	**121,705,290**	**+3,215,795**	**+1.24%**
**Mismatch ≤ 1**			
**Universal**	**148,988,429**	**0**	**57.63%**
**GSNAP**	**149,178,928**	**+190,499**	**+0.08%**
**Ethnicity-Specific**	**149,101,356**	**+112,927**	**+0.05%**
**RefEdit**	**149,667,323**	**+678,894**	**+0.27%**
**RefEdit+**	**150,162,304**	**+1,173,875**	**+0.46%**
**Mismatch ≤ 2**			
**Universal**	**163,866,521**	**0**	**63.39%**
**GSNAP**	**163,971,831**	**+105,310**	**+0.04%**
**Ethnicity-Specific**	**163,908,578**	**+42,057**	**+0.02%**
**RefEdit**	**164,339,083**	**+472,562**	**+0.18%**
**RefEdit+**	**164,646,915**	**+780,394**	**+0.30%**

It is perhaps not too surprising to see that the usage of RefEdit and RefEdit+ results in improved read mapping rates, since more accurate reference genome information is provided to them. What is important to note here is exactly how much improvement RefEdit and RefEdit+ can achieve and at which sites the mapping rate improvement is achieved. An increase on the order of an additional 1% of reads being mapped is a tremendous gain, generating a significant increase in information content for the researcher. It is additionally useful that the largest gains using RefEdit come when allowing the fewest mismatches. In this way, a researcher can choose to allow one fewer mismatch during mapping while still generating the same mapping rate as he or she would have had with the universal genome. This will help to limit the number of false positive variant calls in the analysis while not reducing the information content. RefEdit allows a researcher the flexibility to ask for more information by keeping the mismatch level the same or ask for fewer false positives while keeping the information level the same. As no two studies are alike, this flexibility is a tremendous benefit of this approach.

### Genotype calling consistency

**Fig 4:**
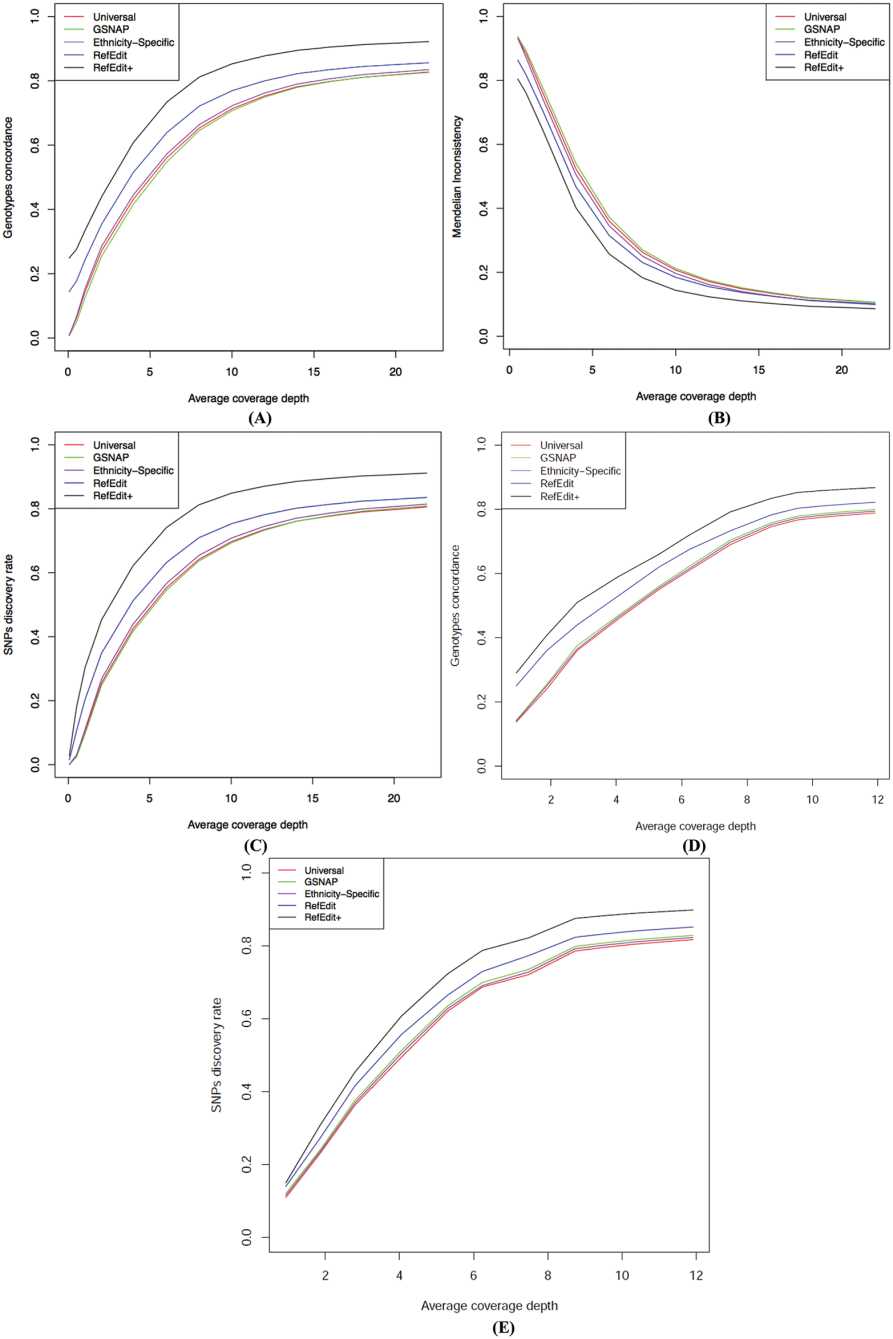
(A) Comparison of genotype calling consistency among the five read mapping strategies for all chromosome 1 SNPs in NA19238 using CGI genotypes as the gold standard. The read length is 36 bp. (B) Comparison of Mendelian Inconsistency among the five read mapping strategies among all chromosome 1 SNPs in the YRI trio (NA19238, NA19239 and NA19240). (C) Comparison of SNP discovery rates among the five read mapping strategies for all chromosome 1 SNPs in NA19238 using CGI genotypes as the gold standard. The read length is 36 bp. (D) Comparison of genotype calling consistency among the five read mapping strategies for all chromosome 1 SNPs in NA19238 using CGI genotypes as the gold standard. The read length is 100 bp. (E) Comparison of SNP discovery rates among the five read mapping strategies for all chromosome 1 SNPs in NA19238 using CGI genotypes as the gold standard. The read length is 100 bp.

Using the CGI genotype calls as the gold standard, we evaluated the genotype calling consistency of RefEdit/RefEdit+ with three competing methods at 13 different sequencing depths (0.5x, 1x, 2x, 4x, 6x, 8x, 10x, 12x, 14x, 16x, 18x, 20x, 22x) on individual NA19238. For each sequencing depth, performance comparison is conducted on the subset of genotypes that are not ref/ref (according to CGI genotypes) that are called by GATK (Genome Analysis Toolkit) [49]. Fig 4A shows the concordance of the non-ref/ref genotypes for five different mapping strategies. As expected, the genotype call consistency improves as the sequencing depth increases. Our RefEdit and RefEdit+ methods consistently outperformed the three competing methods in all read depths, with RefEdit+ performing the best. These results clearly demonstrate that incorporating genotype information of the individual into the read mapping process helps improving the accuracy of genotype calls. Note that the concordance rate is lower than reported elsewhere in the literature [50]. This is because here we chose a lower quality threshold (stand_emit_conf) in GATK to allow inclusion of more SNPs in the performance comparison study in light of the difference in sensitivity of different methods. Using the more commonly used threshold results in higher concordance across board and a similar pattern in terms of performance improvements of RefEdit and RefEdit+.

Remarkably, we discovered that the read mapping using our RefEdit+ strategy can achieve the same level of accuracy as the read mapping using the universal reference genome, by using only a fraction of the reads required by the latter. Fig 4A shows that the method using the universal genome requires a sequencing depth of 22x to reach the same accuracy as RefEdit+ at a sequencing depth of ~9x, albeit with about 4% fewer SNPs called by RefEdit+ at lower sequencing depth (S5 Table). Given the cost associated with the sequencing depth, RefEdit+ provides a key benefit in terms of cost effectiveness. Compared to mapping using the universal reference genome, applying RefEdit+ improve genotype concordance across all sequencing depth tested, for example, from 43% to 61% (4X depth of coverage), from 82% to 92% (20X depth of coverage).

The detailed breakdown table of genotype concordance for five methods can be found in the S6 Table, which shows that RefEdit+ moves a large proportion of genotypes that were previously incorrectly called as ref/ref by other methods to the correct genotypes of ref/alt or alt/alt, according to the CGI genotypes. The main reason for the incorrect ref/ref calls made by using the universal reference genome is that fewer reads that contain the alternative allele can be mapped to the correct locations compared to reads that contain the reference allele.

It has been shown that common and rare variants (MAF ≤ 1%) display different properties [51]. It is therefore of interest to find out whether the level of improvement in genotype calling consistency depends on the MAF of SNPs. We stratified all the SNPs identified by CGI to three categories according to its MAF: MAF ≤ 1%, 1% < MAF ≤ 5% and MAF > 5%. Proportions of genotyped and imputed SNPs in these categories are shown in S7 Table. S8A, S8B and S8C Table is the detailed breakdown tables of genotype concordance of the non-ref/ref genotypes for five different mapping methods in the three categories of SNPs. From these results, we found that improvement in genotype calling consistency is achieved for both rare and common variants. The overall variant calling consistency increases when MAF increases.

### Mendelian inconsistency

A drawback of evaluating performance using genotype concordance as above is that we need to designate a gold standard which may contain errors of its own, although error rate is rather low. Given that there are genotype data from two different platforms (array-based and sequencing-based) for parent-offspring trios from the International HapMap and 1000 Genomes Projects, an alternative metric for performance evaluation is MI which counts the number of loci that show Mendelian errors within the trio. MI has been used in Dewey et al. to evaluate the performance of the ethnicity-specific major allele reference genome approach [48]. For this study, we used data from chromosome 1 of an YRI trio (NA19238, NA19239 and NA19240) to calculate and compare MI at 13 different depths of coverage (0.5x, 1x, 2x, 4x, 6x, 8x, 10x, 12x, 14x, 16x, 18x, 20x, 22x). We only compared performance at loci where all three individuals made the genotype calls and not all of them have homozygous genotypes. MI rates are illustrated in Fig 4B, which shows that the RefEdit+ method has the lowest MI values across all sequencing depths. A breakdown table of MI for all methods at different sequencing depths can be found in S9 Table. Compared to mapping using the universal reference genome, applying RefEdit+ can significantly reduce MI across various sequencing depths: from 52% to 40% (4X depth of coverage), and from 11% to 9% (20X depth of coverage).

### SNP identification

Besides genotype calling consistency at known SNP sites, when conducting WGS studies, it is also important to correctly identify novel SNP variants, as was illustrated in the previous example (Fig 2). Therefore, we assess whether RefEdit+ also improves SNP detection. To be specific, we compared the SNP detection rate when using different read mapping methods at different sequencing depths. For each read mapping strategy, we define the SNP detection rate as follows: among all SNPs identified by CGI sequencing, the proportion of SNPs that are also identified by GATK (non-ref/ref genotypes). As shown in Fig 4C, when the read length is 36 bp RefEdit+ is able to identify the most number of SNPs, followed by RefEdit. The performance enhancement of RefEdit/RefEdit+ is maximized at about 10x coverage.

Next we again stratified all the SNPs identified by CGI into three categories according to their MAFs: MAF ≤ 1%, 1% < MAF ≤ 5% and MAF > 5%. S10A, S10B and S10C Table is the detailed breakdown tables of SNP discovery rates for five different mapping methods in the three categories of SNPs. These results again indicate that using RefEdit and RefEdit+ results in an improved SNP detection rate for both rare and common variants. The best SNP detection rate is achieved for SNPs with 1% < MAF ≤ 5%.

## Impact of longer reads on the above results

In all the analyses conducted above, we set the minimum read length to be 36 bp to utilize all the reads that are being generated. With ever improving sequencing technologies, longer and longer reads are being generated. It is of great interest to know whether the improved read length has any impact on the results we have seen so far. After all, it is possible that increased read length may improve the read mapping and SNP calling such that the benefit of using our RefEdit/RefEdit+ tools is no longer significant. To make this assessment, we repeat all the aforementioned analysis using only reads with 100 bp read length. Here the default mismatch threshold value (5) is used. The results are summarized in S11 Table (read mapping rate), Fig 4D (genotype calling consistency) and Fig 4E (SNP discovery rate). From these results, we observe that for longer reads, RefEdit/RefEdit+ again significantly improves performance over competing read mapping strategies.

## Experimentally phased haplotype as gold standard

As explained earlier, the CGI genotype gold standard inevitably contains errors of its own. For the sake of comprehensiveness, here we choose an alternative gold standard to conduct another round of performance evaluation. In a recent publication, Kuleshov et al. applied a novel long read haplotyping technology to obtain three phased human genomes [52]. We choose the haplotype information provided on the three HapMap samples, NA12892 (mother), NA12891 (father) and NA12878 (child), as the new gold standard. We follow the same procedure described in the previous section. From reads produced by the 1000 Genomes consortium, we specifically choose the 100 bp long reads to test in this study to see how RefEdit and RefEdit+ perform for long read WGS studies. S5 Fig shows the genotype calling consistency of the five read mapping strategies for NA12878. At the sequencing depth 20x, the genotype calling consistencies are summarized in S12 Table. From the figure and table, we see that using the new gold standard, the results again suggests that RefEdit and RefEditor+, especially RefEdit+, produce more accurate genotype calls. The improvement pattern is consistent across the three individuals.

## Impact of different mapping tools

So far, we only use BWA as the read mapping algorithm. To understand whether the mapping tools used will have an impact on the relative performance of RefEdit, we tested another commonly used read mapping algorithm, Bowtie 2 [53]. We use the same procedure described in the previous section except swapping the read mapping tool from BWA to Bowtie 2 to evaluate the genotype calling consistency. From reads produced by the 1000 Genomes consortium, we specifically choose the 100 bp long reads to test performance for long read WGS studies. The genotype calling consistency rates for NA19238 are summarized in S6 Fig. The result again suggests that RefEdit and RefEditor+, especially RefEdit+, produce more accurate genotype calls. The improvement pattern is consistent with the results obtained using BWA as the mapping tool.

## Discussion

With the price of DNA sequencing continuing its rapid decline, whole genome sequencing will likely to be performed *en masse* in research laboratories and perhaps clinics with the primary goal of identifying genetic variants. Mapping the sequencing reads to the human genome is an important early step to analyze data from all sequencing-based experiments including WGS. Multiple studies [47, 48] have demonstrated that genetic variants that occur in about 1% of the genome have a non-ignorable impact on the mapping accuracy, which in turn affects the accuracy of the genotype calls of these variants. Scientists have attempted to address this issue by either incorporating all known genetic variants [47] or ethnicity-specific major alleles [48] into the mapping process. In this study, we go one step further and propose a novel method that takes advantage of the increasingly available personal genotype information. The key of our approach is to customize the reference genome using assayed and imputed genotypes of that individual. Our extensive performance comparison studies demonstrate significant improvement in terms of read mapping, genotype calling and SNP identification.

The performance improvement of RefEditor over existing mapping strategies is easy to understand, because more information is being incorporated. Our work showed that the improvement could be achieved computationally efficiently and in a straightforward fashion using RefEditor. Because array-based genotyping technologies have matured and cost less than WGS, they have been the choice for most large-scale association studies to date [29]. A slew of special-design genotyping chips have also been developed or under-development to supplement the mundane GWAS genotyping chips [54, 55]. As a result, large amount of dense genotyping information is readily available for large cohorts of samples. Many WGS studies were conducted on these samples [56, 57]. Such a design makes our personalized reference genome strategy very attractive.

There are tools available for constructing personal genomes that integrate known variants into the reference genome such as vcf2diploid in AlleleSeq [22] and perEditor [27]. Compared to these existing tools, there are several unique characteristics and contributions of RefEditor. First the purpose of RefEditor is to enable precision variant calling and discovery in large scale, population level studies such as WGS and WES utilizing known individual genotypes. Second, to augment assayed genotypes, we take advantage of the powerful genotype imputation tools to add imputed genotypes into consideration when customizing the reference genome. We found adding imputed genotypes substantially improves the performance of RefEditor. Third, RefEditor does not require phase information; it automatically produces a personalized reference genome index file, which can be fed into any read mapping tools available. Furthermore, read mapping with RefEditor can be carried out on each individual independently in a parallel fashion. Because of this, RefEditor can be easily integrated into an existing WGS or WES analysis pipeline as a module with little modification.

It has been reported in the literature that multi-sample SNP calling strategy improves genotype calling in WGS studies [50]. Since that particular approach is carried out after the read mapping step, our strategy can also be applied during the read mapping step which we believe will further enhance the genotype calling downstream. Due to the requirement of a reasonable number of samples in the cohort to apply the multi-sample calling strategy, we are unable to evaluate the potential performance enhancement under that scenario in the current study.

An important lesson we learned is that the genotype imputation strategy plays a key role in performance improvement for RefEdit+. Genotype imputation has been monumentally successful in GWAS analysis. We demonstrate that high quality imputed genotypes also improve the reference genome customization and therefore produce improved read mapping and genotype calling results.

An extension of our customized reference genome strategy is to apply RefEditor iteratively for multiple rounds. Specifically, after genotypes were called with the help of RefEditor, we can combine these new genotypes with existing (assayed and imputed) genotypes that were used earlier to obtain an updated set of existing genotypes, and then apply RefEditor to perform read mapping and genotype calling again. The same strategy can also be applied to WGS samples without existing genotype information.

Our performance comparison results demonstrate the importance and benefits of incorporating existing genotype information in read mapping, genotype calling and variants discovery in WGS studies. Admittedly, researchers need to spend extra time and effort to perform read mapping with RefEditor: unlike using a single universal reference genome, one has to generate a reference genome for each individual sample in the cohort. A post-process step is also needed after read mapping. However, with our RefEditor package, the whole read mapping process can be automated using simple scripts, and therefore very little human time and intervention is needed in adopting our personalized read mapping strategy. As for computation time, in our experiment on a single core 1.4G Hz CPU and 8GB memory, Diploid Constructor took 4 minutes and 32 seconds to construct the diploid reference genome from hg19.fa and 15,568,754 genotypes (3,900,277 non-ref/ref). The reference genome size increased by 0.2 GB (from 3.0 GB to 3.2 GB) and indexing time increased 5 minutes and 30 seconds (from 87m8s to 92m38s). Read mapping time increased 5 seconds (from 18m49 to 18m54s) to map 5,112,949 reads (read length is 36 bp). Mapping Converter took 49 seconds to convert the intermediate mapping results. Therefore, we believe the overall extra computation cost is quite manageable. Compared to the time spend on collecting sample and sequencing, we believe the extra computation time spent to improve SNP calling accuracy is well justified. Furthermore, for a large cohort, reference genome editing and the subsequent read mapping step can be done in parallel among individuals, which is difficult for multi-sample SNP calling.

It is perhaps not a surprise that a personalized diploid reference genome, incorporating known and imputed genotype of an individual, can result in improved read mapping, and hence more accurate variant calling and discovery. However, until now it is unclear how much improvement this strategy may achieve. By conducting carefully designed, extensive and thorough comparisons, we report that RefEditor, especially when imputed genotypes are added in RefEdit+, will result in substantial improvement in the accuracy of genotype calling and discovery, even with longer read lengths (100 bp). Given the importance of accurately identifying genetic variants and in light of our results, we strongly advocate the adoption of new strategy of using personalized reference genome in population level sequencing-based genetic studies such as WGS and WES.

## Materials and Methods

### RefEdit+ pipeline

The main objective of this project is to construct the personalized diploid reference genome using pre-existing genotype information of an individual, which is typically stored in a Variant Call Format (VCF) file (https://github.com/samtools/hts-specs). This reference genome can then be used for mapping reads generated from any sequencing assay conducted on this individual to improve the read mapping accuracy. There is no need to modify the read mapping software itself. Since genotype information is increasingly available from more and more array-based genotyping and sequencing experiments, we believe incorporating such information in the read-mapping step is important and beneficial. This goal can be conveniently achieved with RefEdit and RefEdit+, with the later contains an additional imputation step to augment the existing genotypes set. The RefEdit+ pipeline consists of the following steps:

#### Step 1 Genotype imputation

In order to increase genotype information that can be used to customize the reference genome, we turn to the genotyping imputation techniques that have been developed in the past five years and showed great success in finding association of untyped SNPs and disease phenotype in many GWAS studies [58, 59]. In this study, we used MaCH version 1.0 [31] and Minimac [36] programs to perform genotype imputation. Default parameters are used for MaCH and Minimac throughout this pipeline. We use population-specific reference panels from the 1000 Genomes Project [41] which contains 25,802,094 SNPs for Yoruba in Ibadan, Nigeria (YRI) and 17,076,866 for Utah residents with ancestry from northern and western Europe (CEU). The two reference panels we used do not contain haplotypes from the two trios we studied, neither do they contain haplotypes from offspring in any trio. We use Rsq threshold of 0.7 for imputation quality control to balance the number of qualified genotypes and quality of imputation.

#### Step 2 Add alternative alleles (genotyped and imputed) to the reference genome

Next, we combine genotyped and imputed genotypes and use them to modify NCBI reference genome 37.1 (HG19 reference) to create a new personalized diploid reference genome. This step is achieved by using the program Diploid Constructor contained in the RefEditor software package. This new reference genome can be fed into any existing mapping tool in the exact same way as the universal reference genome. During the construction process, no action is taken at loci where genotypes are homozygous wild type (reference allele); at loci where genotypes are homozygous mutant alleles we edit the corresponding nucleotides in the reference genome sequence file; at heterozygous loci we add a mini chromosome of length *w* ≥ 2*k* − 1 bp where *k* is the read length. Users can specify their own *w*. When *w* > 2*k* − 1 indels can be better detected at the cost of longer read mapping time. Suggested value of *w* is 2*k* − 1 + 2*m*, where *m* is the maximum allowed indels during read mapping. In all studies presented here, read length *k* is 36, we set *m* to be 2 which is the default indel length used by BWA for read length 36. The sequence of this mini chromosome is identical to the corresponding segment of the universal reference genome except at the middle position in which the alternative allele of that SNP is placed. If two SNPs are located near each other, i.e., with distance of *d* bp, where *d* < *k* + *m*, we create mini chromosomes of all possible combinations of haplotypes that can possibly be covered by a read at the given read length. For two SNPs located far apart, the two alleles on the two mini-chromosomes are not necessarily in phase. For other imputed variants like indels, we modify corresponding mini chromosomes to reflect such type of mutations. Those mini chromosomes are concatenated to the end of each traditional chromosome defined in the reference file, with a sequence of “N”s of *m* + 1 in length to separate them. An auxiliary file is created to record the genomic location of these mini chromosomes. We could let these “mini chromosomes” to stand alone. The reason we choose to ligate them with the original ones is to ensure pair-end read mapping function to work properly because many mapping tools check whether the two ends map to the same chromosome.

RefEditor can also accept an optional command line argument indicating the individual’s gender. When this argument is set for female individuals, chromosome Y will be excluded from the personalized reference genome. Using RefEdit, only non-ref/ref genotypes identified by the genotyping array will be incorporated, whereas using RefEdit+, all non-ref/ref genotypes identified from either the genotyping array or imputation will be incorporated.

#### Step 3 Read mapping using customized diploid reference genome

The customized diploid reference genome can be treated the same as the universal reference genome and used by almost all existing read mapping software. For this study, we use BWA v0.5.9 [6] with default parameters for its high performance on short reads mapping. The raw output of the mapping step needs to be post-processed such that reads mapped to those mini chromosomes are correctly interpreted as mapped to the corresponding genomic locations. Correspondingly, the mapping quality scores of these reads will also be reassigned according to the Phred-scaled probability of mismatches between the read and reference [60]. The conversion is necessary because these multi-mapped reads (one mapped to the correct genomic location, another one mapped to the mini chromosome which corresponds to the same genomic location) are in fact mapped to a unique location in the reference genome. Hence their low mapping quality score (due to the incorrect “multiple-mapping” assignment) should be converted to a high quality score corresponding to unique mapping. This step is achieved by using the program Mapping Converter contained in RefEditor.

#### Step 4 SNP finding and genotype calling

Genotypes are called from the reads successfully mapped with positive mapping quality found in sorted BAM format file. We use the Genome Analysis Toolkit (GATK) [49] to call genotypes. GATK is a widely used software package for detecting SNPs and calling genotypes from single or multiple samples. It takes into account the quality scores of each base in the mapped reads. The output from GATK will be filtered to only keep SNPs.

### Competing read mapping strategies

Various strategies have been developed for dealing with sequence variants in read mapping. Here we briefly review other competing methods.

#### Ethnicity-specific major allele reference genome

In a recent study, Dewey et al. pointed out that the major alleles at many genomic loci are different among populations [48]. Given this, Dewey et al. developed a novel strategy that creates a set of ethnicity-specific reference genomes, including European, African and East Asian. In these reference genomes, the allele that is most frequent among that particular population is used at polymorphic loci, resulting in around 1.5 million modifications in each population compare to the universal reference genome [48]. Read mapping is then performed against these ethnicity-specific major allele reference genomes. Dewey et al. showed that in real studies, using the ethnicity-specific reference genome results in improvement of genotype calling accuracy for disease-associated variant loci [48].

#### GSNAP

GSNAP uses universal reference genome and all SNPs from dbSNP in mapping. It also uses its own mapping algorithm based on hash tables generated from sampled k-mers from reference genome [47]. GSNAP considers all possible genotypes while still maintains running speed comparable to other existing read-mapping software, which impact the mapping results of 7–8% transcriptional reads although it does not significantly increase mapping success rates [47].

## Supporting Information

S1 Text Directions for downloading data and source code as well as other web resources. (DOCX)

S1 Fig Comparison of genotype called before and after imputation for individuals NA19238 and NA12716. Non-ref/ref genotypes before and after imputation are incorporated into the customized reference genome construction for RefEdit and RefEdit+ methods respectively. (A) Genotype composition before/after imputation for sample NA19238. (B) Genotype composition before/after imputation for sample NA12716. (C) The overlapping of non-ref/ref genotypes between imputation and CGI for sample NA19238. Concordance is 98.94%. (D) The overlapping of non-ref/ref genotypes between imputation and CGI for sample NA12716. Concordance is 98.99%.
(DOCX)

S2 Fig Venn’s diagram illustrating SNPs with genotypes obtained from Affymetrix Axiom array, imputation and CGI sequencing for sample NA19238. (1) There are 4,611,084 overlapping SNPs between Affymetrix Axiom array and CGI with 99.75% concordant rate. (2) There are 6,851,861 overlapping SNPs between imputed and CGI with concordance rate 98.58%. (3) There are 2,965,053 SNPs with imputed genotype but not called by CGI sequencing. (4) There are 20,295,528 SNPs that called by CGI sequencing but not from Affymetrix Axiom array or imputation. Only 321,790 are non-ref/ref genotypes.
(DOCX)

S3 Fig Proportions of genotyped and imputed SNPs at different MAF values. (DOCX)

S4 Fig The proportions of imputed genotypes that passed the threshold and their accuracy compare to CGI gold standard across different Rsq value thresholds. The red curve indicates the concordance between imputed genotypes and CGI after applying the Rsq threshold. The blue curve indicates the proportions of the genotypes that pass the Rsq threshold.
(DOCX)

S5 Fig Comparison of genotype calling consistency among the five read mapping strategies for all chromosome 1 SNPs on NA12878. The read length is 100 bp. The phased VCF files produced by Kuleshov et al. are used as the gold standard.
(DOCX)

S6 Fig Comparison of genotype calling consistency among the five read mapping strategies for all chromosome 1 SNPs on NA19238, using Bowtie2 as the read mapping tool. The read length is 100 bp and the CGI genotypes are used as the gold standard.
(DOCX)

S1 Table The total number and percentages of the three different types of genotypes for SNPs that are being genotyped by the Affymetrix Axiom array, imputed or sequenced by CGI. (DOCX)

S2 Table Genotyping concordance rates for SNPs (including the ref/ref genotypes) that are assayed (by the Affymetrix Axiom array) or imputed (from the genotyped SNPs). Genotypes obtained from CGI sequencing were used as the gold standard.
(DOCX)

S3 Table The total numbers and percentages of the three types of genotypes from SNPs that are both assayed by the Affymetrix Axiom array and called by the CGI sequencing. (DOCX)

S4 Table The total numbers and percentages of the three types of genotypes from SNPs that are both imputed (from SNPs assayed by the Affymetrix Axiom array) and called by the CGI sequencing. (DOCX)

S5 Table Genotype calling (by GATK) consistency comparison of five mapping strategies for NA19238 on chromosome 1. The CGI genotypes are used as the gold standard.
(DOCX)

S6 Table Comparison between GATK genotype calling results among the five mapping strategies and CGI sequencing for NA19238 on chromosome 1. The sequencing depth is 22x. The differences (+/-) are the results of comparing to genotype calls using the universal reference genome method. The RefEdit and RefEdit+ methods increase the concordance (shaded parts) between genotype calls and the CGI gold standard genotypes.
(DOCX)

S7 Table Percentage of genotyped and imputed SNPs at different MAF values. (DOCX)

S8 Table Comparison of genotype calling consistency of five mapping strategies for all chromosome 1 SNPs stratified by different MAFs on NA19238. The CGI genotypes are used as the gold standard.
(DOCX)

S9 Table Comparison of Mendelian inconsistency among the five mapping strategies for all chromosome 1 SNPs in the YRI trio (NA19238, NA19239 and NA19240). The differences (+/-) are the results of comparing with MI using the universal reference genome in the read mapping step.
(DOCX)

S10 Table Comparison of SNP discovery rate among the five mapping strategies for all chromosome 1 SNPs stratified by different MAFs in NA19238. The CGI genotypes are used as the gold standard.
(DOCX)

S11 Table Comparison of read mapping rates among the five read mapping strategies for individual NA19238 (369,013,935 reads) with read length 100 bp. (DOCX)

S12 Table Comparison of genotype calling consistency among the five read mapping strategies on all chromosome 1 SNPs from individuals NA12878, NA12891 and NA 12892 respectively with read length 100bp and sequencing depth at 20x. The phased VCF files produced by Kuleshov et al. are used as the gold standard.
(DOCX)
