# Supplementary material for: One Size Doesn't Fit All - RefEditor: Building Personalized Diploid Reference Genome to Improve Read Mapping and Genotype Calling in Next Generation Sequencing Studies
Source: PLoS Comput Biol. 2015 Aug 12;11(8):e1004448. doi: 10.1371/journal.pcbi.1004448 (PMC4534450; doi:10.1371/journal.pcbi.1004448)
Supplement: S1 Text — (DOCX) [file pcbi.1004448.s001.docx]

**Web Resources**

The URLs for source code and data presented are as follows:

RefEditor source code,

<https://github.com/superyuan/refeditor>

Universal Human Genome (version 19 / NCBI v37) hg19.fa, <http://hgdownload.cse.ucsc.edu/goldenPath/hg19/chromosomes/>

MaCH version 1.0.18, <http://www.sph.umich.edu/csg/abecasis/MaCH/download/mach.1.0.18.source.tgz>

Minimac RELEASE STAMP 2012-11-16,

<http://www.sph.umich.edu/csg/cfuchsb/minimac-beta-2012.11.16.tgz>

Ethnicity specific reference genome, <http://datadryad.org/bitstream/handle/10255/dryad.35120/YRIref.fasta.zip?sequence=3>

All fastq files (release date: 2014-10-18),

[ftp://ftp-trace.ncbi.nih.gov/1000genomes/ftp/data/](ftp://ftp-trace.ncbi.nih.gov/1000genomes/ftp/data/NA19238/sequence_read/)

Affymetrix Axiom array genotypes (v4), <ftp://ftp.1000genomes.ebi.ac.uk/vol1/ftp/technical/working/20110210_Affymetrix_Axiom/Affymetrix_Axiom_DB_2010_v4_b37.vcf.gz>

CGI high coverage sequenced genotypes (v 20130808),

<ftp://ftp-trace.ncbi.nih.gov/1000genomes/ftp/technical/working/20130808_multi_sample_cg_calls/>

Phased resolved VCF files are downloaded from

<http://www.stanford.edu/~kuleshov/NA12878.vcf.gz>

<http://www.stanford.edu/~kuleshov/NA12891.vcf.gz>

<http://www.stanford.edu/~kuleshov/NA12892.vcf.gz>

Reference panel is downloaded from <ftp://share.sph.umich.edu/1000genomes/fullProject/2012.03.14/phase1_release_v3.20101123.snps_indels_svs.genotypes.refpanel.ALL.vcf.gz.tgz>
