# Supplementary figures and images for: One Size Doesn't Fit All - RefEditor: Building Personalized Diploid Reference Genome to Improve Read Mapping and Genotype Calling in Next Generation Sequencing Studies
Source: PLoS Comput Biol. 2015 Aug 12;11(8):e1004448. doi: 10.1371/journal.pcbi.1004448 (PMC4534450; doi:10.1371/journal.pcbi.1004448)

**S1 Fig.**

NA19238 NA12716


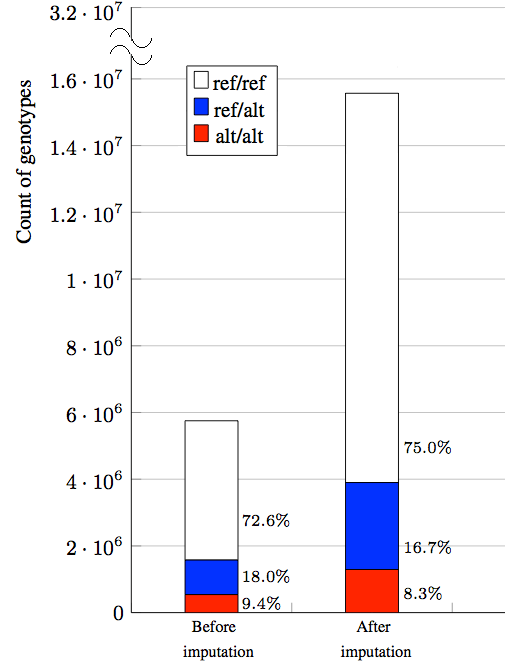

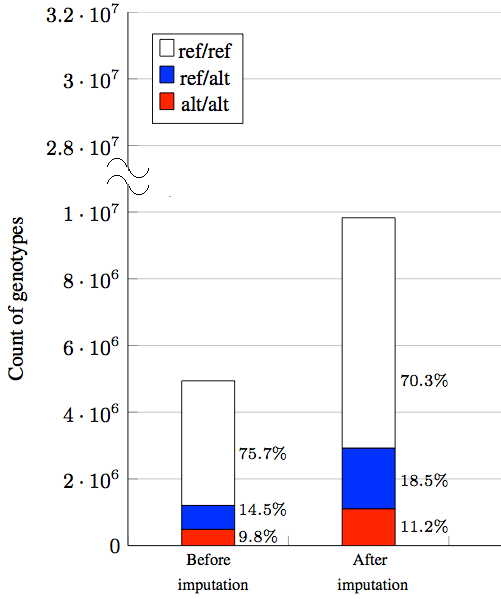


(A) (B)

NA19238 NA12716


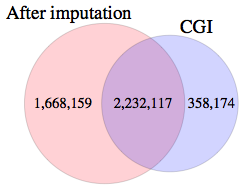

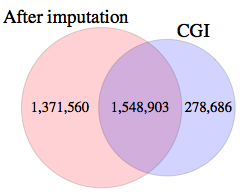


(C) (D)

Supplement: S1 Fig — Non-ref/ref genotypes before and after imputation are incorporated into the customized reference genome construction for RefEdit and RefEdit+ methods respectively. (A) Genotype composition before/after imputation for sample NA19238. (B) Genotype composition before/after imputation for sample NA12716. (C) The overlapping of non-ref/ref genotypes between imputation and CGI for sample NA19238. Concordance is 98.94%. (D) The overlapping of non-ref/ref genotypes between imputation and CGI for sample NA12716. Concordance is 98.99%. (DOCX) [file pcbi.1004448.s002.docx]

**S2 Fig.**


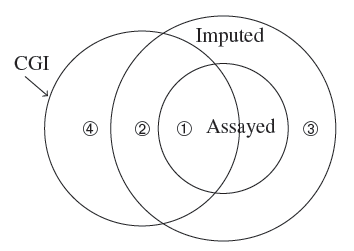

Supplement: S2 Fig — (1) There are 4,611,084 overlapping SNPs between Affymetrix Axiom array and CGI with 99.75% concordant rate. (2) There are 6,851,861 overlapping SNPs between imputed and CGI with concordance rate 98.58%. (3) There are 2,965,053 SNPs with imputed genotype but not called by CGI sequencing. (4) There are 20,295,528 SNPs that called by CGI sequencing but not from Affymetrix Axiom array or imputation. Only 321,790 are non-ref/ref genotypes. (DOCX) [file pcbi.1004448.s003.docx]

**S3 Fig.**


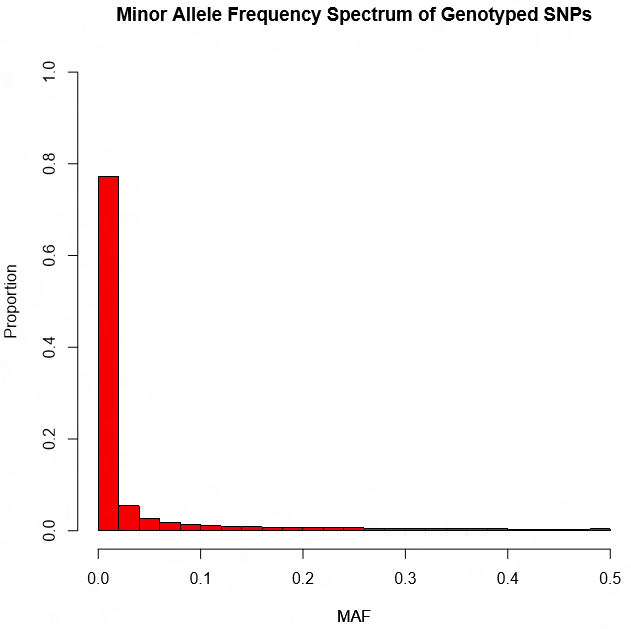


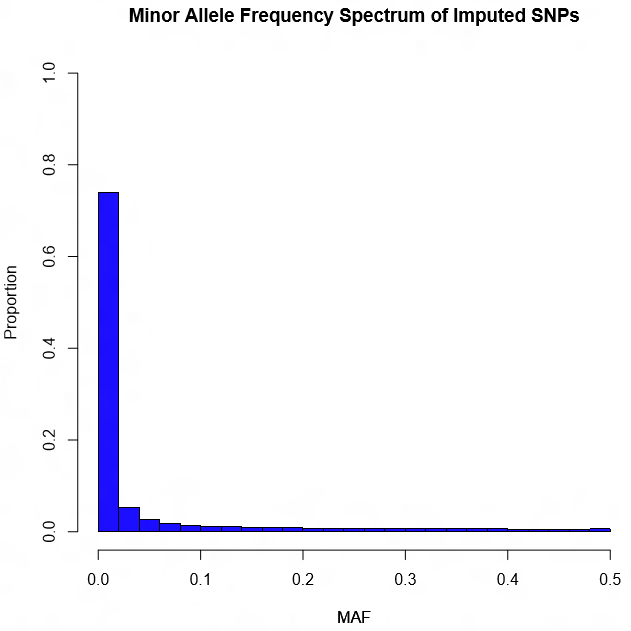

Supplement: S3 Fig — (DOCX) [file pcbi.1004448.s004.docx]

**S4 Fig.**


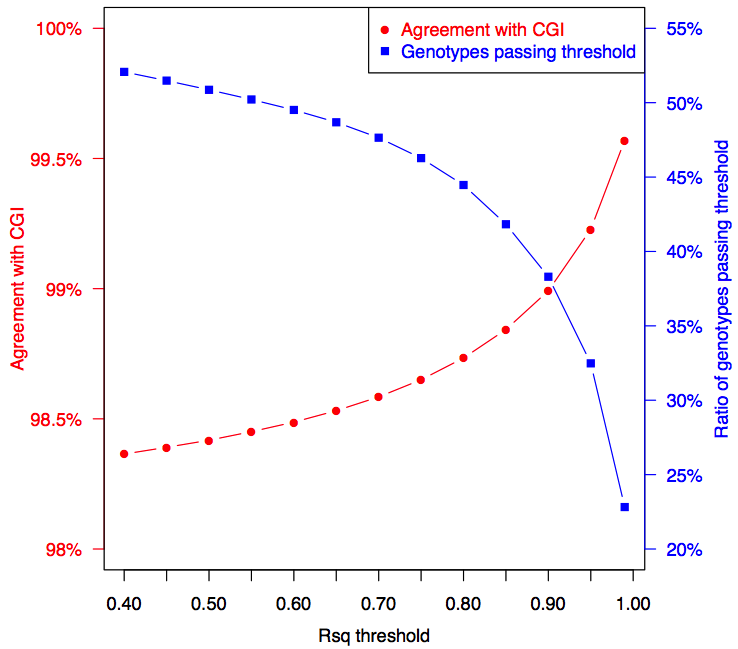

Supplement: S4 Fig — The red curve indicates the concordance between imputed genotypes and CGI after applying the Rsq threshold. The blue curve indicates the proportions of the genotypes that pass the Rsq threshold. (DOCX) [file pcbi.1004448.s005.docx]

**S5 Fig.**


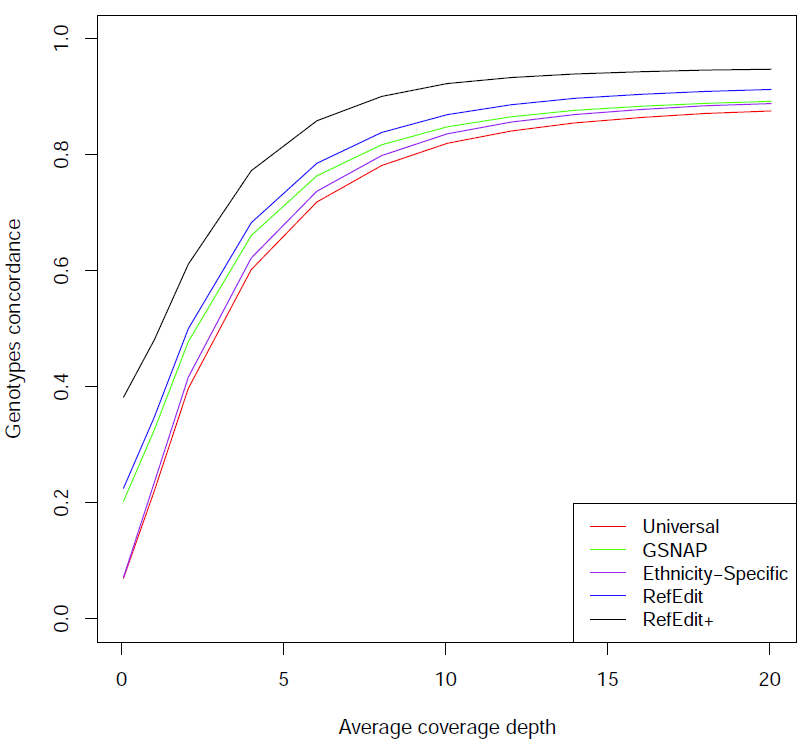

Supplement: S5 Fig — The read length is 100 bp. The phased VCF files produced by Kuleshov et al. are used as the gold standard. (DOCX) [file pcbi.1004448.s006.docx]

**S6 Fig.**


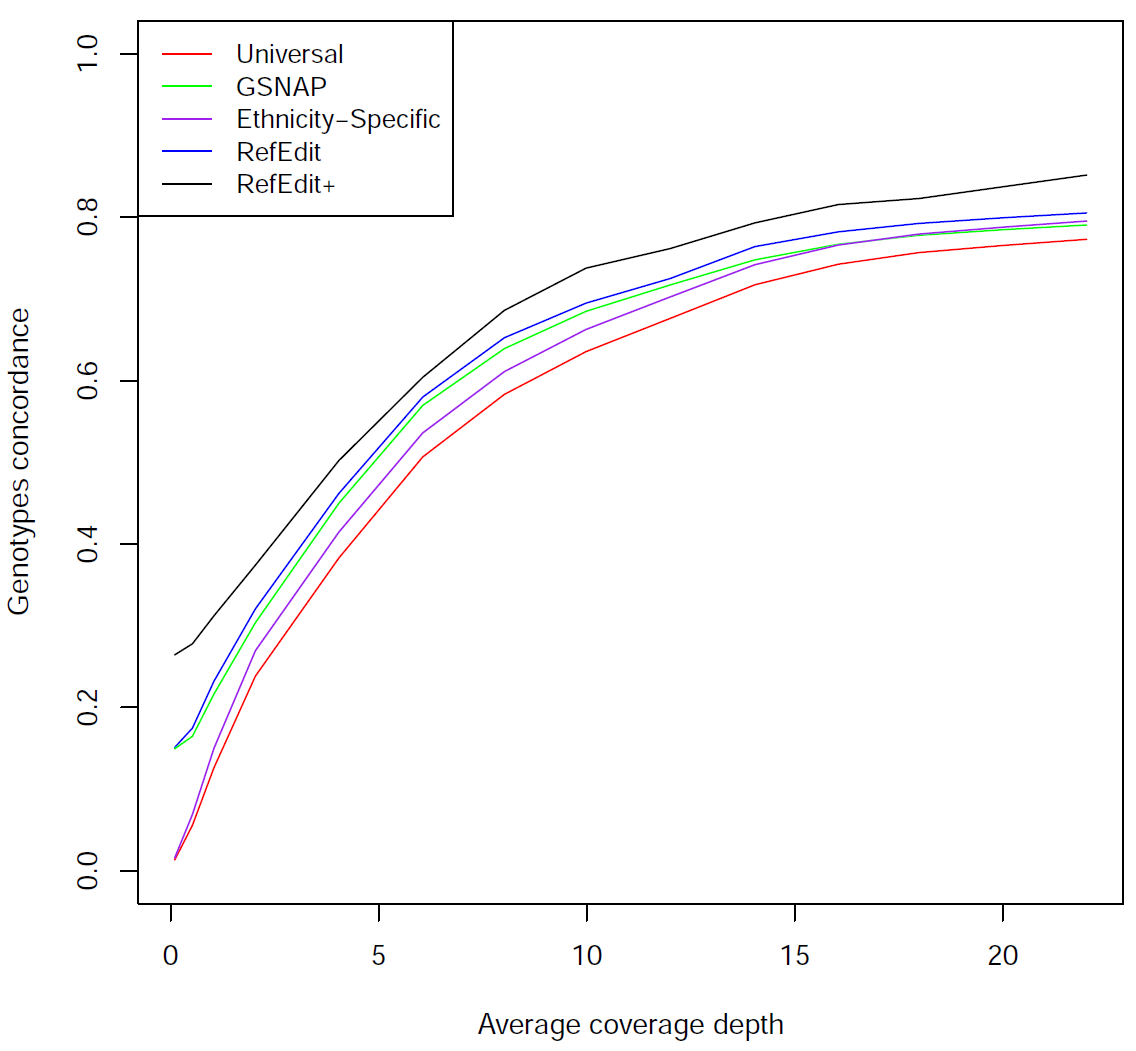

Supplement: S6 Fig — The read length is 100 bp and the CGI genotypes are used as the gold standard. (DOCX) [file pcbi.1004448.s007.docx]
