# Supplementary material for: One Size Doesn't Fit All - RefEditor: Building Personalized Diploid Reference Genome to Improve Read Mapping and Genotype Calling in Next Generation Sequencing Studies
Source: PLoS Comput Biol. 2015 Aug 12;11(8):e1004448. doi: 10.1371/journal.pcbi.1004448 (PMC4534450; doi:10.1371/journal.pcbi.1004448)
Supplement: S1 Table — (DOCX) [file pcbi.1004448.s008.docx]

**S1 Table. The total number and percentages of the three different types of genotypes for SNPs that are being genotyped by the Affymetrix Axiom array, imputed or sequenced by CGI.**

|  | NA19238 | | | | |
| --- | --- | --- | --- | --- | --- |
|  | Before Imputation | | After Imputation | | CGI |
| ref/ref | 4,175,515 | 72.6% | 11,668,478 | 75% | 29,168,182 |
| ref/alt | 1,034,870 | 18.0% | 2,606,560 | 16.7% | 1,957,401 |
| alt/alt | 541,455 | 9.4% | 1,293,716 | 8.3% | 632,890 |
| total | 5,751,840 | | 15,568,754 | | 31,758,473 |
|  | NA12716 | | | | |
|  | Before Imputation | | After Imputation | | CGI |
| ref/ref | 3,735,889 | 75.6% | 6,908,428 | 70.3% | 28,922,009 |
| ref/alt | 718,039 | 14.5% | 1,819,007 | 18.5% | 1,288,470 |
| alt/alt | 486,581 | 9.9% | 1,101,456 | 11.2% | 539,120 |
| total | 4,940,509 | | 9,828,891 | | 30,749,599 |
