# Supplementary material for: One Size Doesn't Fit All - RefEditor: Building Personalized Diploid Reference Genome to Improve Read Mapping and Genotype Calling in Next Generation Sequencing Studies
Source: PLoS Comput Biol. 2015 Aug 12;11(8):e1004448. doi: 10.1371/journal.pcbi.1004448 (PMC4534450; doi:10.1371/journal.pcbi.1004448)
Supplement: S2 Table — Genotypes obtained from CGI sequencing were used as the gold standard. (DOCX) [file pcbi.1004448.s009.docx]

**S2 Table. Genotyping concordance rates for SNPs (including the ref/ref genotypes) that are assayed (by the Affymetrix Axiom array) or imputed (from the genotyped SNPs). Genotypes obtained from CGI sequencing were used as the gold standard.**

|  | Assayed | Imputed |
| --- | --- | --- |
| NA19238 | 99.75% | 99.05% |
| NA19239 | 99.63% | 98.95% |
| NA19240 | 99.80% | 99.08% |
| NA12716 | 99.83% | 99.32% |
| NA12717 | 99.58% | 98.97% |
