# Supplementary material for: One Size Doesn't Fit All - RefEditor: Building Personalized Diploid Reference Genome to Improve Read Mapping and Genotype Calling in Next Generation Sequencing Studies
Source: PLoS Comput Biol. 2015 Aug 12;11(8):e1004448. doi: 10.1371/journal.pcbi.1004448 (PMC4534450; doi:10.1371/journal.pcbi.1004448)
Supplement: S3 Table — (DOCX) [file pcbi.1004448.s010.docx]

**S3 Table. The total numbers and percentages of the three types of genotypes from SNPs that are both assayed by the Affymetrix Axiom array and called by the CGI sequencing.**

Affymetrix genotypes

|  | NA19238 | | | | | |
| --- | --- | --- | --- | --- | --- | --- |
|  | ref/ref | | ref/alt | | alt/alt | |
| ref/alt | 3,539,756 | 76.77% | 4,947 | 0.11% | 637 | 0.01% |
| ref/alt  CGI genotypes | 2,174 | 0.05% | 773,506 | 16.77% | 1,659 | 0.04% |
| alt/alt | 77 | 0.00% | 1,918 | 0.04% | 286,410 | 6.21% |
|  | NA12716 | | | | | |
|  | ref/ref | | ref/alt | | alt/alt | |
| ref/alt | 3,112,334 | 80.59% | 3,316 | 0.09% | 593 | 0.02% |
| ref/alt | 1,079 | 0.03% | 503,822 | 13.05% | 676 | 0.02% |
| alt/alt | 60 | 0.00% | 725 | 0.02% | 239,267 | 6.20% |
