# Supplementary material for: One Size Doesn't Fit All - RefEditor: Building Personalized Diploid Reference Genome to Improve Read Mapping and Genotype Calling in Next Generation Sequencing Studies
Source: PLoS Comput Biol. 2015 Aug 12;11(8):e1004448. doi: 10.1371/journal.pcbi.1004448 (PMC4534450; doi:10.1371/journal.pcbi.1004448)
Supplement: S4 Table — (DOCX) [file pcbi.1004448.s011.docx]

**S4 Table. The total numbers and percentages of the three types of genotypes from SNPs that are both imputed (from SNPs assayed by the Affymetrix Axiom array) and called by the CGI sequencing.**

Imputed genotypes

|  | NA19238 | | | | | |
| --- | --- | --- | --- | --- | --- | --- |
|  | ref/ref | | ref/alt | | alt/alt | |
| ref/ref | 5,606,214 | 81.82% | 42,481 | 0.62% | 409 | 0.01% |
| ref/alt  CGI genotypes | 33,710 | 0.49% | 880,603 | 12.85% | 12,417 | 0.18% |
| alt/alt | 423 | 0.01% | 7,569 | 0.11% | 268,035 | 3.91% |
|  | NA12716 | | | | | |
|  | ref/ref | | ref/alt | | alt/alt | |
| ref/ref | 2,305,314 | 73.50% | 12,779 | 0.41% | 606 | 0.02% |
| ref/alt | 13,482 | 0.43% | 569,413 | 18.15% | 11,216 | 0.36% |
| alt/alt | 76 | 0.00% | 2,933 | 0.09% | 220,851 | 7.04% |
