# Supplementary material for: One Size Doesn't Fit All - RefEditor: Building Personalized Diploid Reference Genome to Improve Read Mapping and Genotype Calling in Next Generation Sequencing Studies
Source: PLoS Comput Biol. 2015 Aug 12;11(8):e1004448. doi: 10.1371/journal.pcbi.1004448 (PMC4534450; doi:10.1371/journal.pcbi.1004448)
Supplement: S5 Table — The CGI genotypes are used as the gold standard. (DOCX) [file pcbi.1004448.s012.docx]

**S5 Table. Genotype calling (by GATK) consistency comparison of five mapping strategies for NA19238 on chromosome 1. The CGI genotypes are used as the gold standard.**

| **Coverage** | **Genotypes*** | **Universal** | **GSNAP** | **Ethnicity** | **RefEdit** | **RefEdit+** |
| --- | --- | --- | --- | --- | --- | --- |
| 0.5 | 58,278 | 6.17% | 4.90% | 6.60% | 17.66% | 27.57% |
| 1 | 94,144 | 14.39% | 12.61% | 15.39% | 24.34% | 33.46% |
| 2 | 129,059 | 27.10% | 25.44% | 28.53% | 35.35% | 43.87% |
| 4 | 156,834 | 43.08% | 41.56% | 44.43% | 51.44% | 60.70% |
| 6 | 172,097 | 56.07% | 54.84% | 57.25% | 63.88% | 73.37% |
| 8 | 179,626 | 65.31% | 64.56% | 66.37% | 72.12% | 81.14% |
| 10 | 183,037 | 71.34% | 70.78% | 72.30% | 76.95% | 85.30% |
| 12 | 184,963 | 75.31% | 74.97% | 76.22% | 80.00% | 87.75% |
| 14 | 186,298 | 78.16% | 77.98% | 78.99% | 82.22% | 89.46% |
| 16 | 187,174 | 79.78% | 79.74% | 80.63% | 83.48% | 90.49% |
| 18 | 187,931 | 81.11% | 81.15% | 81.96% | 84.47% | 91.26% |
| 20 | 188,320 | 81.88% | 81.96% | 82.70% | 85.04% | 91.72% |
| 22 | 188,755 | 82.64% | 82.79% | 83.47% | 85.61% | 92.19% |

*indicates the total number of SNPs on chromosome 1 with non-ref/ref genotypes called by all five strategies and the CGI sequencing.
