# Supplementary material for: One Size Doesn't Fit All - RefEditor: Building Personalized Diploid Reference Genome to Improve Read Mapping and Genotype Calling in Next Generation Sequencing Studies
Source: PLoS Comput Biol. 2015 Aug 12;11(8):e1004448. doi: 10.1371/journal.pcbi.1004448 (PMC4534450; doi:10.1371/journal.pcbi.1004448)
Supplement: S6 Table — The sequencing depth is 22x. The differences (+/-) are the results of comparing to genotype calls using the universal reference genome method. The RefEdit and RefEdit+ methods increase the concordance (shaded parts) between genotype calls and the CGI gold standard genotypes. (DOCX) [file pcbi.1004448.s013.docx]

**S6 Table. Comparison between GATK genotype calling results among the five mapping strategies and CGI sequencing for NA19238 on chromosome 1. The sequencing depth is 22x. The differences (+/-) are the results of comparing to genotype calls using the universal reference genome method. The RefEdit and RefEdit+ methods increase the concordance (shaded parts) between genotype calls and the CGI gold standard genotypes.**

|  |  | ref/ref | ref/alt | alt/alt |
| --- | --- | --- | --- | --- |
| ref/ref | Universal | 2,098,021 | 406 | 27 |
|  | Ethnicity-Specific | -1 | -1 | +2 |
| CGI genotypes Gold Standard | GSNAP | -33 | -10 | +43 |
|  | RefEdit | -64 | +50 | +14 |
|  | RefEdit+ | -220 | +145 | +75 |
| ref/alt | Universal | 27,349 | 113,138 | 1,637 |
|  | Ethnicity-Specific | -1,296 | +938 | +358 |
|  | GSNAP | -370 | +445 | -75 |
|  | RefEdit | -5,269 | +5,095 | +174 |
|  | RefEdit+ | -18,294 | +15,456 | +2,838 |
| alt/alt | Universal | 2,907 | 408 | 42,857 |
|  | Ethnicity-Specific | -496 | -128 | +624 |
|  | GSNAP | -255 | +421 | -166 |
|  | RefEdit | -617 | +59 | +558 |
|  | RefEdit+ | -2,510 | -264 | +2,774 |
