# Supplementary material for: One Size Doesn't Fit All - RefEditor: Building Personalized Diploid Reference Genome to Improve Read Mapping and Genotype Calling in Next Generation Sequencing Studies
Source: PLoS Comput Biol. 2015 Aug 12;11(8):e1004448. doi: 10.1371/journal.pcbi.1004448 (PMC4534450; doi:10.1371/journal.pcbi.1004448)
Supplement: S7 Table — (DOCX) [file pcbi.1004448.s014.docx]

**S7 Table. Percentage of Genotyped and Imputed SNPs at different MAF values.**

|  | Genotyped | Imputed |
| --- | --- | --- |
| MAF≤1% | 69.58% | 66.46% |
| 1%<MAF≤5% | 14.61% | 14.35% |
| MAF>5% | 15.81% | 19.19% |
