# Supplementary material for: One Size Doesn't Fit All - RefEditor: Building Personalized Diploid Reference Genome to Improve Read Mapping and Genotype Calling in Next Generation Sequencing Studies
Source: PLoS Comput Biol. 2015 Aug 12;11(8):e1004448. doi: 10.1371/journal.pcbi.1004448 (PMC4534450; doi:10.1371/journal.pcbi.1004448)
Supplement: S8 Table — The CGI genotypes are used as the gold standard. (DOCX) [file pcbi.1004448.s015.docx]

**S8 Table. Comparison of genotype calling consistency of five mapping strategies for all chromosome 1 SNPs stratified by different MAFs on NA19238. The CGI genotypes are used as the gold standard.**

(A) MAF≤1%

| Coverage | Universal | GSNAP | Ethnicity | RefEdit | RefEdit+ |
| --- | --- | --- | --- | --- | --- |
| 0.5 | 5.61% | 4.44% | 6.02% | 15.85% | 24.90% |
| 1 | 13.33% | 11.58% | 14.17% | 22.38% | 30.91% |
| 2 | 25.79% | 23.99% | 27.07% | 33.31% | 41.38% |
| 4 | 40.41% | 38.67% | 41.60% | 48.27% | 56.77% |
| 6 | 53.07% | 52.62% | 55.19% | 61.42% | 70.12% |
| 8 | 63.29% | 62.76% | 64.14% | 69.90% | 78.99% |
| 10 | 69.09% | 69.09% | 70.67% | 74.99% | 83.13% |
| 12 | 72.89% | 72.72% | 73.15% | 77.09% | 84.95% |
| 14 | 76.21% | 76.15% | 76.58% | 80.20% | 86.81% |
| 16 | 77.36% | 77.10% | 78.04% | 80.79% | 87.79% |
| 18 | 78.93% | 78.34% | 79.10% | 82.17% | 88.46% |
| 20 | 79.48% | 80.00% | 80.92% | 82.79% | 88.98% |
| 22 | 79.44% | 80.79% | 81.41% | 82.41% | 89.32% |

(B) 1%<MAF≤5%

| Coverage | Universal | GSNAP | Ethnicity | RefEdit | RefEdit+ |
| --- | --- | --- | --- | --- | --- |
| 0.5 | 6.12% | 4.92% | 6.62% | 17.61% | 27.57% |
| 1 | 13.32% | 11.69% | 14.34% | 22.52% | 31.18% |
| 2 | 26.65% | 25.03% | 28.21% | 34.71% | 43.11% |
| 4 | 42.76% | 41.15% | 43.96% | 50.66% | 59.82% |
| 6 | 56.39% | 55.46% | 57.83% | 65.34% | 74.46% |
| 8 | 66.64% | 65.47% | 66.82% | 72.89% | 82.38% |
| 10 | 72.28% | 71.70% | 74.51% | 79.26% | 87.26% |
| 12 | 78.04% | 77.76% | 79.76% | 83.56% | 91.01% |
| 14 | 81.40% | 82.36% | 83.05% | 86.92% | 93.97% |
| 16 | 82.68% | 82.79% | 84.93% | 87.01% | 94.42% |
| 18 | 84.36% | 84.43% | 84.89% | 87.44% | 95.01% |
| 20 | 84.18% | 85.83% | 86.34% | 87.74% | 95.12% |
| 22 | 85.64% | 85.40% | 86.03% | 88.75% | 95.26% |

(C) MAF>5%

| Coverage | Universal | GSNAP | Ethnicity | RefEdit | RefEdit+ |
| --- | --- | --- | --- | --- | --- |
| 0.5 | 7.97% | 6.34% | 8.43% | 23.41% | 36.02% |
| 1 | 17.58% | 15.65% | 18.96% | 30.15% | 40.96% |
| 2 | 31.25% | 29.98% | 33.04% | 41.79% | 51.72% |
| 4 | 50.43% | 49.56% | 52.31% | 60.40% | 71.77% |
| 6 | 62.26% | 59.23% | 61.32% | 68.38% | 79.72% |
| 8 | 69.38% | 68.33% | 71.44% | 76.99% | 85.56% |
| 10 | 76.67% | 74.66% | 75.26% | 80.72% | 89.83% |
| 12 | 79.92% | 79.11% | 82.02% | 85.39% | 93.03% |
| 14 | 81.77% | 80.47% | 83.42% | 85.05% | 94.30% |
| 16 | 83.91% | 84.31% | 84.32% | 87.89% | 94.67% |
| 18 | 85.17% | 86.99% | 88.19% | 89.05% | 96.76% |
| 20 | 86.86% | 84.82% | 85.20% | 89.37% | 96.93% |
| 22 | 89.49% | 86.61% | 87.49% | 92.37% | 98.10% |
|  |  |  |  |  |  |
