# Supplementary material for: One Size Doesn't Fit All - RefEditor: Building Personalized Diploid Reference Genome to Improve Read Mapping and Genotype Calling in Next Generation Sequencing Studies
Source: PLoS Comput Biol. 2015 Aug 12;11(8):e1004448. doi: 10.1371/journal.pcbi.1004448 (PMC4534450; doi:10.1371/journal.pcbi.1004448)
Supplement: S9 Table — The differences (+/-) are the results of comparing with MI using the universal reference genome in the read mapping step. (DOCX) [file pcbi.1004448.s016.docx]

**S9 Table. Comparison of Mendelian Inconsistency among the five mapping strategies for all chromosome 1 SNPs in the YRI trio (NA19238, NA19239 and NA19240). The differences (+/-) are the results of comparing with MI using the universal reference genome in the read mapping step.**

| **Coverage** | **Universal** | **GSNAP** | **Ethnicity** | **RefEdit** | **RefEdit+** |
| --- | --- | --- | --- | --- | --- |
| 0.5 | 93.20% | +0.48% | +0.03% | -6.84% | -12.76% |
| 1 | 88.38% | +0.89% | -1.22% | -6.65% | -12.33% |
| 2 | 76.19% | +1.58% | -1.89% | -5.63% | -11.63% |
| 4 | 52.35% | +1.72% | -1.82% | -5.64% | -12.19% |
| 6 | 36.00% | +1.23% | -1.49% | -4.51% | -10.30% |
| 8 | 26.27% | +0.76% | -1.21% | -3.20% | -7.97% |
| 10 | 20.68% | +0.51% | -1.04% | -2.24% | -6.31% |
| 12 | 17.11% | +0.38% | -0.95% | -1.60% | -4.77% |
| 14 | 14.84% | +0.28% | -0.86% | -1.16% | -3.79% |
| 16 | 13.27% | +019% | -0.81% | -0.88% | -3.12% |
| 18 | 11.95% | +0.12% | -0.67% | -0.78% | -2.58% |
| 20 | 11.26% | -0.04% | -0.52% | -0.74% | -2.23% |
| 22 | 10.59% | -0.09% | -0.43% | -0.70% | -1.96% |
