# Supplementary material for: One Size Doesn't Fit All - RefEditor: Building Personalized Diploid Reference Genome to Improve Read Mapping and Genotype Calling in Next Generation Sequencing Studies
Source: PLoS Comput Biol. 2015 Aug 12;11(8):e1004448. doi: 10.1371/journal.pcbi.1004448 (PMC4534450; doi:10.1371/journal.pcbi.1004448)
Supplement: S10 Table — The CGI genotypes are used as the gold standard. (DOCX) [file pcbi.1004448.s017.docx]

**S10 Table. Comparison of SNP discovery rate among the five mapping strategies for all chromosome 1 SNPs stratified by different MAFs in NA19238. The CGI genotypes are used as the gold standard.**

(A) MAF≤1%

| Coverage | Universal | GSNAP | Ethnicity | RefEdit | RefEdit+ |
| --- | --- | --- | --- | --- | --- |
| 0.5 | 2.36% | 2.90% | 3.03% | 10.57% | 17.87% |
| 1 | 8.89% | 9.99% | 10.65% | 19.89% | 29.01% |
| 2 | 24.01% | 25.24% | 26.82% | 34.39% | 43.81% |
| 4 | 38.57% | 42.60% | 43.46% | 50.38% | 59.85% |
| 6 | 48.94% | 50.12% | 52.05% | 57.52% | 65.87% |
| 8 | 58.46% | 59.32% | 59.61% | 66.63% | 74.59% |
| 10 | 65.63% | 66.30% | 68.00% | 72.42% | 80.63% |
| 12 | 69.90% | 70.50% | 71.87% | 74.06% | 83.25% |
| 14 | 71.51% | 73.03% | 74.13% | 77.87% | 84.80% |
| 16 | 72.63% | 74.15% | 75.02% | 78.36% | 85.75% |
| 18 | 75.38% | 75.44% | 76.25% | 79.22% | 86.13% |
| 20 | 75.88% | 76.85% | 76.98% | 79.70% | 86.24% |
| 22 | 76.71% | 77.00% | 79.71% | 80.45% | 87.05% |

(B) 1%<MAF≤5%

| Coverage | Universal | GSNAP | Ethnicity | RefEdit | RefEdit+ |
| --- | --- | --- | --- | --- | --- |
| 0.5 | 2.38% | 2.96% | 3.10% | 10.90% | 18.70% |
| 1 | 8.81% | 9.68% | 10.25% | 18.84% | 27.87% |
| 2 | 24.12% | 26.21% | 27.36% | 35.09% | 45.92% |
| 4 | 38.84% | 41.01% | 41.23% | 48.38% | 60.00% |
| 6 | 51.12% | 53.57% | 53.94% | 60.29% | 70.76% |
| 8 | 67.73% | 67.87% | 68.74% | 76.46% | 78.99% |
| 10 | 69.60% | 71.12% | 72.64% | 77.62% | 80.58% |
| 12 | 73.07% | 74.01% | 75.52% | 78.27% | 86.57% |
| 14 | 78.84% | 79.49% | 79.86% | 84.55% | 91.34% |
| 16 | 80.79% | 81.23% | 84.26% | 89.24% | 95.88% |
| 18 | 81.30% | 82.09% | 84.62% | 89.60% | 98.34% |
| 20 | 81.88% | 83.61% | 85.05% | 92.85% | 98.99% |
| 22 | 83.32% | 84.77% | 85.27% | 93.00% | 99.06% |

(C) MAF>5%

| Coverage | Universal | GSNAP | Ethnicity | RefEdit | RefEdit+ |
| --- | --- | --- | --- | --- | --- |
| 0.5 | 2.20% | 2.78% | 2.92% | 10.72% | 18.19% |
| 1 | 11.63% | 12.88% | 13.31% | 23.55% | 36.86% |
| 2 | 25.51% | 26.38% | 26.62% | 36.24% | 49.59% |
| 4 | 46.15% | 47.06% | 47.34% | 55.82% | 70.99% |
| 6 | 71.61% | 72.00% | 73.29% | 78.22% | 83.20% |
| 8 | 73.82% | 77.02% | 78.65% | 80.66% | 84.73% |
| 10 | 75.87% | 81.28% | 81.43% | 83.01% | 86.74% |
| 12 | 80.95% | 82.29% | 82.38% | 84.44% | 88.80% |
| 14 | 81.04% | 83.48% | 84.54% | 85.97% | 89.52% |
| 16 | 84.20% | 86.55% | 87.98% | 88.42% | 91.96% |
| 18 | 84.73% | 86.88% | 88.75% | 89.80% | 92.96% |
| 20 | 85.16% | 90.04% | 90.19% | 91.38% | 94.45% |
| 22 | 90.52% | 90.76% | 91.72% | 92.15% | 96.65% |
