# Supplementary material for: One Size Doesn't Fit All - RefEditor: Building Personalized Diploid Reference Genome to Improve Read Mapping and Genotype Calling in Next Generation Sequencing Studies
Source: PLoS Comput Biol. 2015 Aug 12;11(8):e1004448. doi: 10.1371/journal.pcbi.1004448 (PMC4534450; doi:10.1371/journal.pcbi.1004448)
Supplement: S11 Table — (DOCX) [file pcbi.1004448.s018.docx]

**S11 Table. Comparison of read mapping rates among the five read mapping strategies for individual NA19238 (369,013,935 reads) with read length 100 bp.**

|  | NA19238 | Read length=100 |  |
| --- | --- | --- | --- |
|  | Mapped reads | Difference | Mapping rates |
| Mismatch ≤ 5 |  |  |  |
| Universal | 320,328,206 | 0 | 86.81% |
| GSNAP | 320,806,109 | +477,903 | +0.13% |
| Ethnicity-Specific | 320,644,007 | +315,801 | +0.09% |
| RefEdit | 320,836,533 | +508,327 | +0.14% |
| RefEdit+ | 321,474,347 | +1,146,141 | +0.31% |
