# Supplementary material for: One Size Doesn't Fit All - RefEditor: Building Personalized Diploid Reference Genome to Improve Read Mapping and Genotype Calling in Next Generation Sequencing Studies
Source: PLoS Comput Biol. 2015 Aug 12;11(8):e1004448. doi: 10.1371/journal.pcbi.1004448 (PMC4534450; doi:10.1371/journal.pcbi.1004448)
Supplement: S12 Table — The phased VCF files produced by Kuleshov et al. are used as the gold standard. (DOCX) [file pcbi.1004448.s019.docx]

**S12 Table. Comparison of genotype calling consistency among the five read mapping strategies on all chromosome 1 SNPs from individuals NA12878, NA12891 and NA 12892 respectively with read length 100bp and sequencing depth at 20x. The phased VCF files produced by Kuleshov et al. are used as the gold standard.**

|  | Universal | GSNAP | Ethnicity | RefEdit | RefEdit+ |
| --- | --- | --- | --- | --- | --- |
| NA12878 | 88.30% | 88.75% | 88.77% | 90.33% | 93.70% |
| NA12891 | 88.62% | 89.11% | 89.07% | 90.52% | 93.98% |
| NA12892 | 86.80% | 86.99% | 87.36% | 89.14% | 92.35% |
